# Effect of Sodium Silicate and Sodium Hydroxide Ratios on Compressive Strength of Ceramic Brick and Metakaolin Waste-Based Geopolymer Binder

**DOI:** 10.3390/ma18214947

**Published:** 2025-10-29

**Authors:** Martynas Statkauskas, Danutė Vaičiukynienė, Audrius Grinys, Diana Bajare

**Affiliations:** 1 Faculty of Civil Engineering and Architecture, Kaunas University of Technology, Studentu st. 48, LT-51367 Kaunas, Lithuania; 2 Institute of Sustainable Building Materials and Engineering Systems, Faculty of Civil and Mechanical Engineering, Riga Technical University, 6A Kipsalas, LV-1048 Riga, Latvia

**Keywords:** geopolymer binder, ceramic brick waste, metakaolin waste, sodium silicate, sodium hydroxide, Na_2_SiO_3_/NaOH ratio

## Abstract

The construction industry’s growth is causing a surge in CO_2_ emissions, driven by increased demand for concrete and other building materials. There is a growing demand for more sustainable building materials, including alkali-activated materials. This study investigates the impact of varying ratios of Na_2_SiO_3_ and NaOH on the mechanical properties and microstructure of metakaolin (MKW) and ceramic brick waste (CBW) based geopolymer binder. Geopolymer binder precursors were made of three main CBW/MKW ratios: 100/0%wt. (C100), 50/50%wt. (C50M50), and 0/100%wt. (M100). Alkaline activator solutions had three different Na_2_SiO_3_/NaOH ratios: 0.5, 1.0, and 2.0. The investigation into the geopolymer binder mechanical properties was conducted using a range of analytical methods, including compressive strength, scanning electron microscopy (SEM), X-ray diffraction (XRD), and Fourier transform infrared spectroscopy (FT-IR). The findings of the study indicate that the Na_2_SiO_3_/NaOH ratio alone is inadequate for evaluating geopolymer mechanical properties when different AS/P ratios are employed, given its influence on other parameters, such as the W/S ratio and the total Na_2_O content. CBW-based geopolymer binders demonstrate limited capacity to attain substantial compressive strengths because they contain high amounts of unreacted CBW particles, as shown by XRD analysis. The incorporation of MKW precursor resulted in enhanced reactivity and intensified geopolymerization reaction. After the evaluation of all essential ratios, the most favorable Na_2_SiO_3_/NaOH ratio is 1.0. This determination was based on the highest strengths observed in designs that contained ≥50% of MKW precursor, attributed to predominance of goosecreekite and N-A-S-H gels, as evidenced by XRD and FT-IR analysis.

## 1. Introduction

A substantial body of research has established that Portland cement (OPC) is a prevalent construction material, accounting for a significant proportion of global CO_2_ emissions [1]. The primary component of Portland cement is clinker, which is produced through the combustion of a blend of limestone and clay in rotary kilns at a temperature of 1450 °C. The combustion of one ton of clinker, a major component of cement, has been shown to emit approximately 850 kg of the greenhouse gas known as carbon dioxide [2]. The composition of clinker emission consists of up to 70% of the CO_2_ attributed to the decarbonation of limestone, around 20–30% is the result of the combustion of fuel, and the residual amount is associated with the production processes [3]. As indicated by the statistics presented in the article by Bărbulescu et al. [4], the global cement production demonstrated a considerable augmentation between 1995 and 2024, escalating from 1.39 billion metric tons to over 4 billion tons. This growth reached a peak in 2021 and again in 2023, with production levels reaching 4.4 billion tons in both years. It is evident that global cement production has increased at an alarming rate in recent decades. The cement manufacturing process has been a significant contributor to environmental pollution and climate change, releasing pollutants such as particulate matter (PM), nitrogen oxides (NO_x_), sulfur dioxide (SO_2_), and ammonia (NH_3_) [5]. Furthermore, carbon dioxide (CO_2_) has been identified as the primary contaminant resulting from the accelerated manufacturing of Portland cement, constituting approximately 8% of global emissions [6].

Recent studies have demonstrated that a promising approach for mitigating the carbon dioxide (CO_2_) emissions generated by the cement industry entails the utilization of alternative binding materials. Geopolymers, initially developed by Davidovits, have emerged as a compelling substitute, offering a multitude of advantages. These include advantageous mechanical properties and thermal stability, reduced carbon dioxide emissions, cost-effectiveness, and exceptional thermal and chemical stability [7]. Geopolymers are defined as inorganic polymers that have the capacity to utilize alkali substances, along with natural and waste products, as the primary raw materials during the synthesis process [8]. Geopolymers are the result of geopolymerization, which is the exothermic chemical reaction involving aluminosilicate precursors (powder form) and an alkaline activator (liquid or solid form). The efficacy of the geopolymerization reaction directly correlates with the likelihood of obtaining optimal geopolymer material properties, though too high a level of geopolymerization can lead to adverse properties [9]. Conventionally, the process of geopolymerization entails the hydration and polymerization of its precursors, resulting in a three-dimensional framework of silicon-O and aluminon-tetrahedra. These units are systematically interconnected, characterized by the shared oxygen atoms [10]. The internal structure of geopolymers is influenced by numerous factors, including the source of aluminum and silicon in the precursors, the ratio of silicon to aluminum oxides, the fineness of the precursor, the type of alkaline solution, the concentration level of the solution, and the mixing/curing regime [11].

Metakaolin waste is a pervasive by-product on a global scale, yet its potential applications remain to be fully explored. Currently, metakaolin has been found to be significantly more environmentally friendly than cement. It is already frequently utilized in the ceramics industry, along with a partial replacement of cement in the concrete industry. Metakaolin is obtained through a calcination process in which kaolinite is subjected to temperatures between 700 and 800 °C. During this process, kaolinite undergoes a hydroxylation reaction, which causes it to lose water [12]. This material is abundant in aluminum and silicon, thus one promising avenue for its utilization involves its incorporation into the development of geopolymer binders. In recent decades, ceramic waste has been the subject of extensive research as an industrial by-product. Zhang et al. [13] distinguishes a few ceramic waste categories: sanitary waste (toilets, sinks), tableware (plates, cups, bowls), ceramic tiles (wall, floor and roof tiles), and ceramic bricks. The primary sources of these materials included defective production, utilization, or construction and demolition of buildings. The disposal of ceramic waste materials was typically conducted through two primary methods: direct burial in the ground or placement in an open landfill. Ceramic waste possesses considerable potential for use in geopolymer development due to its high aluminum and silicon content and pozzolanic activity [14]. Therefore, the accumulation of ceramic waste contaminates the environment, necessitating the urgent recycling of this waste as geopolymers; we present a viable solution to address this issue.

Researchers worldwide have investigated the mechanical properties of geopolymer composites, focusing on the impact of different alkaline solutions and their ratios. The type of alkaline solution employed exerts a substantial influence on the geopolymerization reaction, thereby affecting the development of mechanical strength. The activator necessitates the presence of an alkaline compound in an aqueous form. These include hydroxides (Na^+^, K^+^, and Ca^2+^), silicates (Na^+^ and K^+^), carbonates, and sulfates [15]. The selection of the activator is contingent upon two primary factors: reactivity and cost. Consequently, the most prevalent activators are silicates and hydroxides, as well as their combinations. As posited by da Silva et al. [16], the utilization of metakaolin as a precursor has been demonstrated to yield a geopolymer morphology that is heterogeneous, porous, and microcracked when employing alkaline sodium silicate and sodium hydroxide. This phenomenon is attributed to rapid activation, thereby resulting in a substantial mechanical strength that can reach up to 80 MPa. According to Ivanović et al. [17], the utilization of Na_2_SiO_3_ and NaOH (2, 4, 6, and 8 M) in conjunction with a Na_2_SiO_3_/NaOH ratio of 1.6 provides a means to ascertain the influence of increasing NaOH concentration on geopolymerization. The outcomes of this investigation indicate that a geopolymer with a more compact structure and higher compressive strength is obtained as the NaOH concentration in the activation solution increases. Petrus et al. [18] studied how the Na_2_SiO_3_/NaOH weight ratio affects the compressive strength of silicate and bentonite-based GPC. They found that increasing the ratio from 1 to 1.5 increases strength and raising it from 1.5 to 2.5 decreases it significantly. Görhan and Kürklü [19] investigated a geopolymer using class F fly ash with varying NaOH (SH) molarities (3 M, 6 M, and 9 M) in conjunction with a sodium silicate (SS) solution. The ratio of sodium silicate to sodium hydroxide (SS:SH) was constant at 2.0. The optimal geopolymer composition was achieved with 6 M NaOH, resulting in the highest compressive strength of 22 MPa, attained after curing at 65 °C for 24 h. Doğan-Sağlamtimur et al. [20] conducted a geopolymer study using fly ash as the precursor and two different activators (Na_2_SiO_3_ and 12 M NaOH), varying the SS:SH solution ratio (1.0, 1.5, 2.0, 2.5, and 3.0). They evaluated the geopolymer’s mechanical properties. The study’s findings show that the strongest compressive strength (76 MPa) was achieved after 28 days when the SS:SH ratio was 2 and the curing regime was set at 70 °C for 24 h. However, as demonstrated in the study by Morsy et al. [21], the highest strength for a low calcium fly ash geopolymer that is cured at 80 °C is attained when the SS:SH ratio is 1.0. Cao et al. [22] conducted a study that examined a blended geopolymer system composed of metakaolin and fly ash, as well as potassium hydroxide. The findings indicate that this geopolymer possesses both substantial compressive strength and notable high-temperature resistance, attributable to the presence of substantial quantities of reactive Al_2_O_3_ in metakaolin. This promotes the formation of silica-aluminate gels, thereby enhancing the geopolymer’s microstructure and compressive strength.

This study develops and characterizes a novel geopolymer binder system made of ceramic brick waste and metakaolin, activated with an alkaline solution. It contributes to the search for sustainable alternatives to conventional Portland cement by valorizing industrial waste materials and exploring their potential in geopolymer formulations. This research addresses a significant gap in the literature regarding the use of ceramic brick waste and metakaolin as individual precursors in alkali-activated binder systems. There is a lack of systematic investigation into their behavior when used alone or together.

The objective of this study is to provide experimental data regarding the impact of varying ratios of sodium silicate and sodium hydroxide (Na_2_SiO_3_/NaOH) on the mechanical properties and microstructure of ceramic brick, and metakaolin waste-based geopolymer binders.

## 2. Materials and Experimental Techniques

### 2.1. Initial Materials

The geopolymer binder is made of three constituents: metakaolin waste, ceramic brick waste, and phosphogypsum. Ceramic brick waste (CBW) is a major type of construction and demolition waste, found in open-air landfills. Samples were crushed and milled to create a powder with a larger surface area. The oxide composition of CBW mainly consists of 70.49% SiO_2_ and 13.24% Al_2_O_3_. CBW is predominantly quartz (66.96%), with smaller amounts of microcline (12.08%), albite (9.47%), and amorphous (9.34%) phases. The mean particle diameter is 12.89 μm, the specific density is 2.77 g/cm^3^, and the fineness is 497.96 m^2^/kg. Metakaolin waste (MKW), formed during the production of expanded glass granules, is a waste by-product of kaolinite clay calcination. It is contaminated with expanded glass particles, resulting in a distinct oxide composition (high Na_2_O concentration) compared to conventional metakaolin. MKW is 55.28% SiO_2_, 30.90% Al_2_O_3_, and 7.46% Na_2_O. It is mostly kaolinite (10.42%), anorthite (8.03%), muscovite (5.31%), and amorphous (73.07%). The mean particle diameter is 11.31 μm, the specific density is 2.59 g/cm^3^, and the fineness is 472.75 m^2^/kg. Phosphogypsum (PG) is produced when making orthophosphoric acid, which fertilizer companies typically make. The oxide composition of PG consists of 56.74% SO_3_ and 39.16% CaO. PG is predominantly composed of basanite and brushite. The mean particle diameter is 11.01 μm, the specific density is 2.51 g/cm^3^, and the fineness is 516.32 m^2^/kg.

The geopolymer binder compositions were designed with three different amounts of ceramic brick and metakaolin waste precursor: 100/0%wt. (C100), 50/50%wt. (C50M50) and 0/100%wt. (M100). The phosphogypsum was utilized as a calcium source, with a constant dosage of 5%wt (weight percent). According to the findings of a previous study [23], this accelerates the geopolymerization reaction and leads to faster setting and improved geopolymer compressive strength. The acceleration is attributed to the presence of calcium oxide in phosphogypsum, which has been demonstrated to enhance the early-stage reaction kinetics of the geopolymer matrix. Alkaline solutions were prepared with three different sodium-silicate-to-sodium-hydroxide (SS/SH) ratios of 0.5, 1.0 and 2.0. The Na_2_SiO_3_ solution was composed of 9.3% Na_2_O, 28% SiO_2_ and 62.7% H_2_O. The 10 M NaOH solution was prepared by blending sodium hydroxide pellets (97% purity) with distilled water.

The present study examined a total of nine geopolymer binder compositions composed of the previously mentioned initial materials and their essential ratios (see Table 1). Metakaolin waste precursor is characterized with a higher reaction rate than ceramic brick waste precursor due to its greater concentration of Al_2_O_3_ and its higher content of amorphous phase. This characteristic enables the reactivity of a greater number of components during the initial stage of geopolymer curing. Consequently, there is an observed variation in the alkaline solution contents across all designed geopolymer binder compositions until the geopolymerization reaction reaches a state of optimal efficiency (C100—AS/P: 0.36; C50M50—AS/P: 0.54; M100—AS/P: 0.83). The variation in alkaline solution contents and SS/SH ratios resulted in alterations to not only Na_2_O content but also to essential ratios such as water-to-solids (W/S) and H_2_O/Na_2_O, as well as to the fresh mixture consistency. Therefore, the SS/SH ratios were carefully selected to ensure the consistency of the mixture composition across the designed range without the addition of supplementary water. The consistency of geopolymer binders was measured using the Vicat apparatus and a plunger, TESTING Bluhm & Feuerherdt, Berlin, Germany. The consistency was maintained throughout all compositions by ensuring the distance between the plunger and baseplate remained constant at (20 ± 5) mm. Moreover, it was observed that higher amounts of sodium silicate tend to thicken the fresh paste, while a higher content of sodium hydroxide tends to liquify the paste.

**Figure 1:**
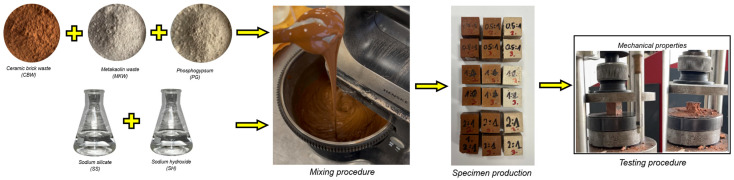
Geopolymer production scheme.

The geopolymer mixing procedure (Figure 1) was prepared using an electric mixer (Philips HR3740/00, Philips, Kaunas, Lithuania) with a power output of 450 watts. The procedure began with the amalgamation of two alkaline activators for a duration of one minute, concurrently ensuring adequate integration of the dry precursors. Subsequently, a three-minute wet mixing process was initiated, during which a liquid alkaline solution was poured onto the dry precursors. The total mixing time was precisely four minutes. The homogeneously mixed geopolymer paste was poured into cubic silicone (20 mm × 20 mm × 20 mm) molds and compacted on a vibrating table to avoid the formation of air bubbles. The molds were concealed with polypropylene to inhibit early moisture loss. The geopolymer binder specimens were placed in an ambient temperature environment (20 ± 2 °C, 60% RH) for 24 h. After rest, an oven curing regime (60 °C for 24 h) was used to enhance the geopolymer binder’s early strength. Specimens were left at room temperature until 7 and 28 days, at which point mechanical properties were tested.

A total of ten specimens were formed for each of the designed geopolymer binder compositions for the mechanical property testing procedures. Five of these specimens were used for testing after 7 days, and the remaining five after 28 days of hardening.

### 2.2. Experimental Techniques

The precursor materials and hardened geopolymer binder specimens were subjected to a series of analyses, which comprised the following tests.

X-ray fluorescence (XRF). The elemental compositions of the precursor materials were determined through the use of XRF analysis. Laboratory apparatus: XRF spectrometer Bruker X-ray S8Tiger WD (Bruker AXS, Karlsruhe, Germany) with Rh tube up to 60 eV.

Laser diffraction. The essential parameters (i.e., particle size distribution, density, and specific surface area) of the precursor materials were established through the use of laser diffraction (dry method). Laboratory apparatus: The CILAS 1090 LD (Cilas, Orléans, France) laser scattering particle size analyzer.

X-ray diffraction (XRD). The mineral composition of the precursor materials and geopolymer binder was identified through XRD analysis. Laboratory apparatus: diffractometer “D8 Advance” (Bruker AXS, Bruker, Karlsruhe, Germany) operating on 40 kV and 40 mA. The X-ray beam was filtered with a 0.02 mm Ni filter to select the CuKα wavelength. The powder X-ray diffraction patterns were identified in accordance with the recommendations of the PDF-2 database. The material was analyzed quantitatively using the Rietveld method (TOPAS 4.1).

Fourier transform infrared spectroscopy (FT-IR). The mineralogical validity of geopolymer products resulting from a synthesis was ascertained through the implementation of FT-IR. Laboratory apparatus: The PerkinElmer FT-IR System spectrometer (PerkinElmer, Waltham, MA, USA). One milligram of the material was mixed with 200 milligrams of potassium bromide (KBr), and the mixture was compressed in a forming press under vacuum to allow for the infrared analysis.

Scanning electron microscopy (SEM). The microstructures of precursor materials and geopolymerization reaction products were examined through the application of SEM. Laboratory apparatus: The FEI Quanta 200 FEG high-performance scanning electron microscope (FEI, Hillsboro, OR, USA) with a Schottky field emission gun (Hitachi, Tokyo, Japan). The chemical composition was subsequently analyzed with a Bruker Quad 5040 EDS detector (123 eV), Bruker, Karlsruhe, Germany.

**Figure 2:**
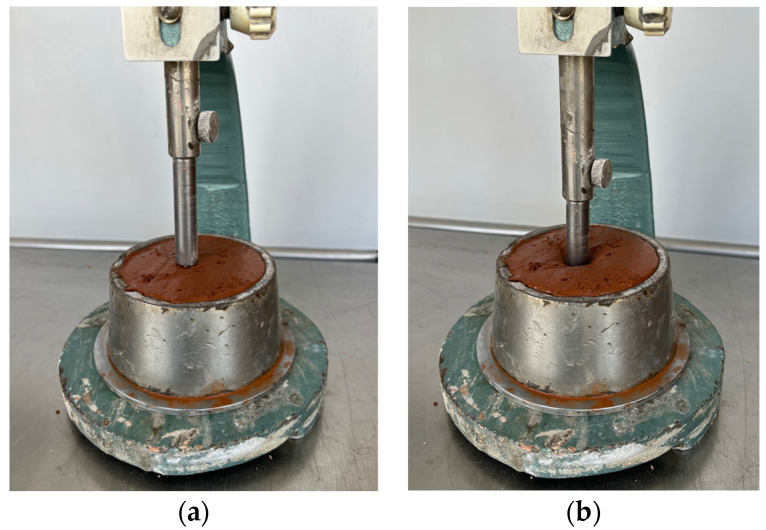
Standard consistency test with Vicat apparatus: (**a**) before penetrating paste, and (**b**) after penetrating paste.

Vicat apparatus (plunger). The consistency of geopolymer binders were determined by standard consistency method according to EN 196-3 [24] with Vicat apparatus and plunger (see Figure 2). Laboratory apparatus: Vicat apparatus with movable rod and a plunger (TESTING Bluhm & Feuerherdt, Berlin, Germany) (10 mm diameter, 50 mm length).

Compressive strength. The mechanical properties of hardened geopolymer binders were evaluated through the implementation of compressive strength performance tests, in accordance with the established standard EN 12390-3 [25]. The testing of specimens was conducted after 7 and 28 days of curing. Laboratory apparatus: Zwick Z100 universal analysis machine (ZwickRoell, Ulm, Germany), with a loading rate of 0.6 ± 0.2 MPa/s.

## 3. Results

### 3.1. Compressive Strength

**Figure 3:**
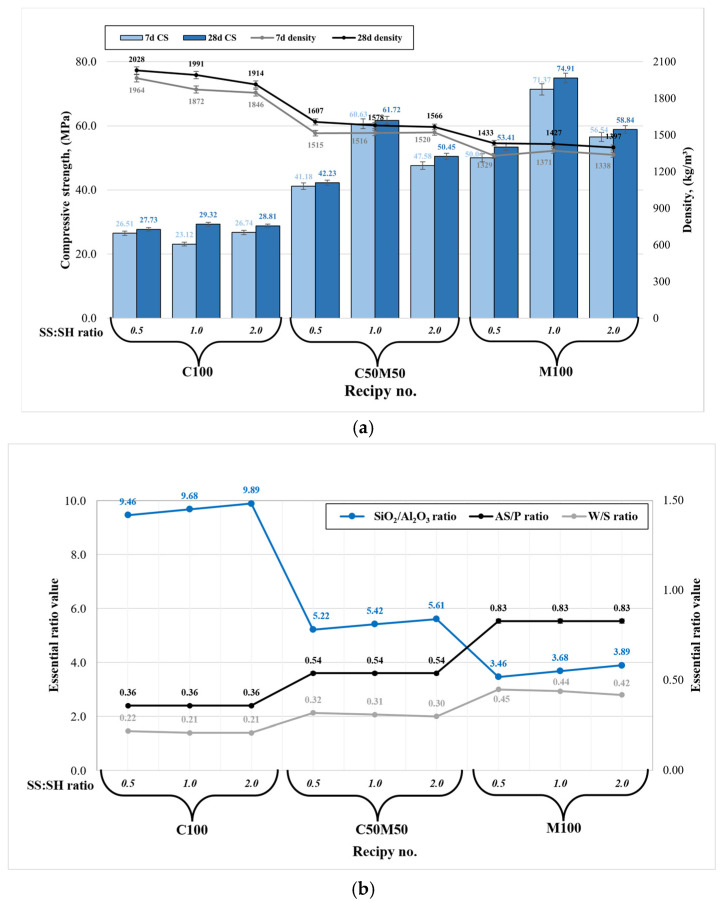
Geopolymer binder mechanical properties: (**a**) compressive strength and density correlation; (**b**) the relationship between the essential ratios.

The compressive strength and material density are the most significant mechanical properties for geopolymer composites, particularly in the context of civil engineering applications. The density of each specimen group (composition) has exhibited variability across different amplitudes. As illustrated in Figure 3a, an increase in metakaolin waste content results in diminished densities, attributable to the presence of lighter MKW particles. Also, higher contents of sodium silicate have been observed to result in diminished densities.

The essential findings of analogous studies with similar precursors and their combinations, in conjunction with disparate parameters of alkaline activators, are summarized in Table 2 [26,27,28,29,30,31,32,33]. Density is closely related to compressive strength: lower density values result in higher compressive strength (see Figure 3a). The maximum compressive strength values (74.9 MPa after 28 days) were recorded for specimens composed exclusively of metakaolin waste with a sodium silicate to sodium hydroxide ratio of 1:1 (M100 1.0). This increase can be attributed to the high reactivity of metakaolin waste, which contains a significant amount of amorphous phase. Metakaolin waste-based geopolymer composition (M100 1.0) has been demonstrated to exhibit high reactivity. However, it has been observed that this composition contains a significantly higher alkaline-solution-to-precursor ratio (AS/P) in comparison to other compositions (see Figure 3b). This observation results in a geopolymer system that contains a higher Na_2_O content of 11.21% and a water-to-solids (W/S) ratio of 0.44. A high amount of Na_2_O has been shown to stimulate faster dissolution in the early stage, which in turn leads to much more intensive geopolymerization reaction when compared with the remaining compositions. However, an elevated W/S ratio engenders superfluous high open porosity due to the abundant presence of free water within the binder matrix. This, in turn, results in material brittleness, as evidenced by the compressive strength test procedure. Therefore, geopolymer binders of this nature exhibit high compressive strength; however, they are less robust in terms of other factors, including durability, water resistance, fire resistance, and freeze/thaw resistance. Consequently, the high reactivity in question is responsible for a robust geopolymerization reaction, which in turn leads to the formation of new hydration products such as goosecreekite. This results in an extremely compact microstructure and, consequently, the highest compressive strength [21]. A comparison of the present study with literature in which metakaolin waste was used as the sole geopolymer precursor [29] reveals a substantial enhancement in strength, with a twofold increase observed in the present study. Also, Chen et al. [34] found that the SiO_2_/Al_2_O_3_ ratio greatly affects metakaolin-based geopolymer mechanical performance, with the ideal ratio ranging from 3.5 to 4.0, similarly to our composition M100 1.0 ratio (see Figure 3b). Furthermore, while the disparity in strength is not substantial, superior outcomes are achieved with higher concentrations of sodium silicate (SS/SH = 2.0) compared to sodium hydroxide (SS/SH = 0.5). The observed phenomenon can be attributed not only to the SS/SH ratio, but also to the elevated content of Na_2_O and free water derived from the NaOH solution. Consequently, this results in a reduction in strength and an increased likelihood of efflorescence due to the excessive Na_2_O content.

An analysis was conducted on the compressive strength outcomes of composition C100, which utilized ceramic brick waste as the sole precursor. The study concluded that the SS/SH ratio does not exert a significant influence on strength, as the strength results ranged from 27.7 to 29.3 MPa (C100 0.5 to C100 2.0) after 28 days. The compositions containing exclusively ceramic brick waste exhibit an elevated SiO_2_/Al_2_O_3_ ratio, reaching approximately 9.5. This indicates an excess of silica in the precursor material. Thus, ceramic brick waste powder (9.34% amorphous phase) exhibits a lower degree of reactivity in comparison to metakaolin waste (73.07% amorphous phase). For this reason, geopolymerization reaction is much slower and as there are lots of unreacted silica, ultimately leading to a much lower compressive strength. Consequently, the geopolymerization reaction is characterized by a significant reduction in velocity, resulting in a substantial accumulation of unreacted silica. This, in turn, engenders a substantial decline in compressive strength. Also, the composition containing a higher amount of NaOH (C100 0.5) showed a higher level of efflorescence. However, compositions exclusively comprising ceramic brick waste exhibited significantly higher densities, ranging from 1914 to 2028 kg/m^3^. This observation signifies that the material possesses a compact microstructure, replete with substantial quantities of unreacted compounds and latent unused potential. As demonstrated in our previous study [35], elevated temperature curing has the potential to stimulate secondary geopolymerization, thereby enhancing the compressive strength of ceramic brick waste-based geopolymer up to 100%. Nevertheless, a comparison of the findings with those of other researchers [31,32] indicates that ceramic brick waste used exclusively as a geopolymer precursor yields compressive strength results that are nearly identical.

The compressive strength outcomes of composition C50M50 1.0 had a SiO_2_/Al_2_O_3_ of around 5.5, which incorporated a blend of ceramic brick waste and metakaolin waste, exhibited optimal compressive strength of 61.7 MPa when the SS/SH ratio was set at 1.0. This geopolymer composition was almost as strong as the best composition (strength of >74 MPa). The enhancement can be attributed to a more balanced ratio of reactive silica and alumina along with lower AS/P and W/S ratios derived from the precursors and alkaline activators. This finding indicates that the addition of significant quantities of metakaolin waste is essential to achieve substantial geopolymer binder compressive strength. Furthermore, a comparable outcome in terms of compressive strength was observed when compared to the study conducted by Sarkar and Dana [33]. However, an excess of metakaolin waste has been observed to result in the thickening of the fresh geopolymer paste, thereby necessitating greater amounts of water for the efficient handling of the fresh material. However, this must be considered with caution, as an increase in alkaline solution or free water content has been shown to disrupt the essential AS/P and W/S ratios and Na_2_O content. This disruption can, in turn, result in undesired geopolymer binder properties. One potential solution to the challenges associated with the incorporation of metakaolin waste precursor into the geopolymer system, particularly in regard to paste mixing, involves the selection of an extended mixing time while maintaining constant AS/P and W/S ratios, in conjunction with the SS/SH ratio. Also, a potential avenue for enhancing the performance of geopolymer binders involves the incorporation of reinforcing materials or fibers. A study by Furtos et al. [36] employed basalt fibers, also referred to as “Minibars,” which have been shown to markedly enhance the mechanical properties of geopolymer composites.

In the pursuit of optimized mechanical properties, it is advised to employ a Na_2_SiO_3_/NaOH ratio that does not fall below 1.0 and does not exceed 2.0, in conjunction with a SiO_2_/Al_2_O_3_ ratio ranging from 3.5 to 5.5. Concurrently, prior to designing a fresh mixture consistency, it is imperative to evaluate the AS/P and W/S ratios, in conjunction with the Na_2_O content, as these parameters exert a substantial influence on the mechanical properties of the hardened geopolymer. It is hypothesized that this approach will yield optimal mechanical strength characteristics when the ceramic brick and metakaolin waste precursors are used solely or as a blend.

### 3.2. Mineralogy and Microstructure

**Figure 4:**
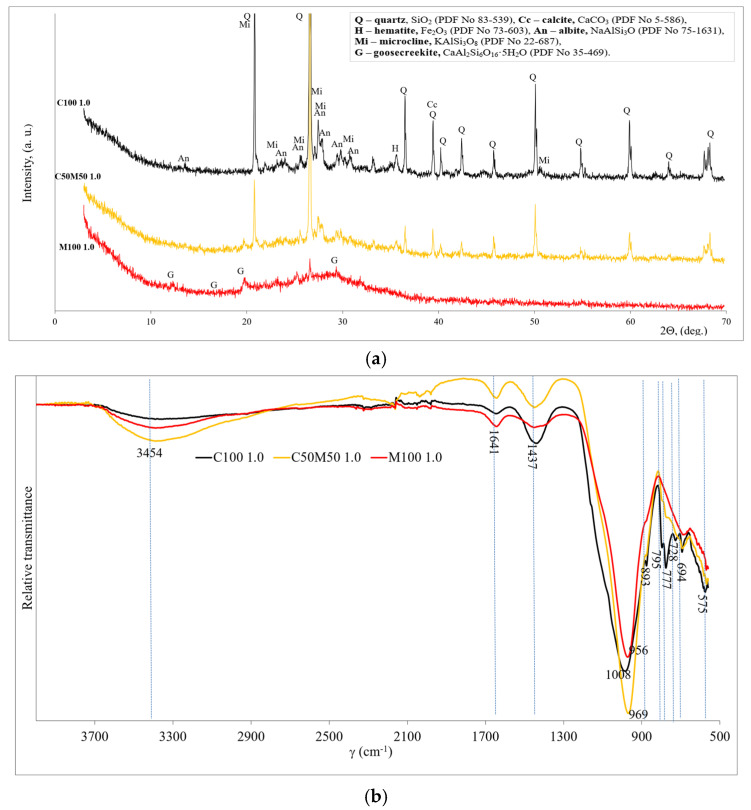
The mineralogy patterns of geopolymer binder (compositions C100 1.0, C50M50 1.0 and M100 1.0): (**a**) XRD; (**b**) FT-IR.

The three samples with the highest compressive strength were selected for the determination of mineral composition which is shown in Figure 4a. All the samples contained quartz left over from the alkali activation of the precursors. When precursors consist of CBW (C100 1.0 and C50M50 1.0), in addition to quartz and microcline, hematite and albite have been detected as crystalline compounds. All these compounds are from CBW, and they do not react after alkali activation. As indicated by the findings of other researchers [37], the presence of analogous residual crystalline phases (quartz, albite, and anorthite) has been detected in blends of recycled brick and recycled ceramic tiles subsequent to alkaline activation. No new crystalline chemical compounds were detected after alkali activation. The XRD pattern of the alkali-activated MKW shows the formation of calcium aluminum silicate hydrate (goosecreekite). Marques et al. [38] detected goosecreekite, which is described as a zeolite compound, in the mineral composition of alkali-activated binders produced on the basis of metakaolin. A hump between 17° and 35°, 2θ diffraction angle (hump centered at 29°), shows amorphous nature compounds (aluminosilicate gel) indicating geopolymerization products dominating in the M100 1.0 sample. In addition, Provis et al. [39] and Sarkar [33] found that an amorphous hump centered at 28° confirms the geopolymerization of the binders.

Figure 4b shows and compares the FT-IR spectra of the three highest strength samples. The bands around 3454 and 1641 cm^−1^ are associated with OH^−^ (probably water) vibrations [40]. In addition, the bands at 1437 and 893 cm^−1^ are associated with the C-O stretching vibration which indicated that calcite and sodium carbonate compounds are due to weathering. The quartz (vibrations of Si-O) is related to the FT-IR bands at about 795, 777, and 694 cm^−1^ [41]. The remaining unreacted quartz and calcite are from the initial materials (CBW). The C100 sample has the highest intensities of quartz and calcite bands, followed by the C50M50 and M100 samples, as MKW contains only traces of quartz and no calcite.

The main board bands are detected in the range 956–1008 cm^−1^ and are related to the asymmetric stretching vibrations of the Si–O–Si/Al in geopolymer structure. The shift to lower wavenumbers may be due to the incorporation of aluminum in the matrix and the higher degree of geopolymerization of the sample. This shift in the bands at 956–1008 cm^−1^ to lower frequencies indicates the creation of new geopolymerization products, according to Rajini et al. [42]. So, by increasing the MKW in the system, it led to a higher degree of geopolymerization [43]. The formation of C-A-S-H and/or N-A-S-H is related to this peak. The lowest intensity of these bands was found in sample M100, as a certain amount of C-A-S-H and/or N-A-S-H gel recrystallizes into a crystalline-like zeolite. Both these hydration products had a positive effect on the development of strength.

**Figure 5:**
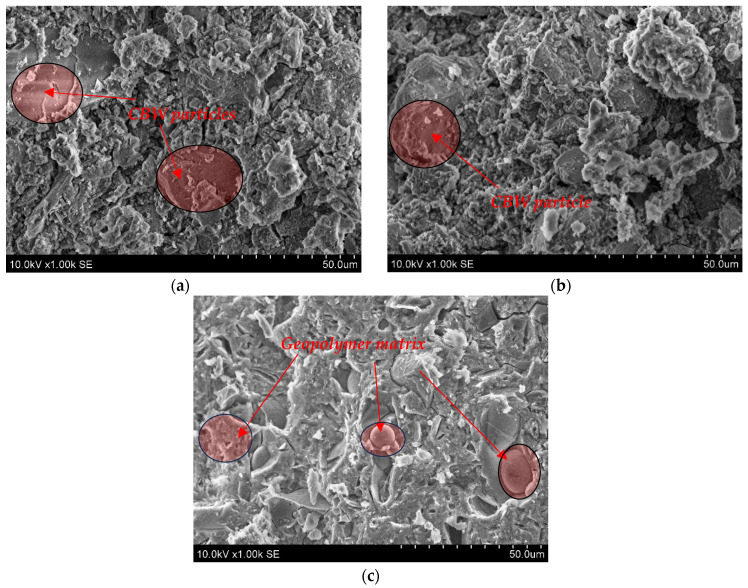
The microstructure images of geopolymer binder (**a**)—C100 1.0; (**b**)—C50M50 1.0; (**c**)—M100 1.0.

The microstructure (SEM analysis) of geopolymer binder based on alkali-activated MKW and CBW after 28 days is shown in Figure 5. Some unreacted CBW particles are detected after alkali activation in the C100 1.0 and C50M50 1.0 samples. These CBW particles were embedded in the aluminosilicate gel matrix as geopolymerization product.

A compact microstructure was observed in the transition zone between the CBW particle and the aluminosilicate gel matrix, and no microcracks were detected. Allaoui et al. [44] found that ceramic sanitary ware waste and geopolymer gel have a similar compact microstructure. A sufficient amount of geopolymer gel and unreacted CBW particles result in relatively high strength of 60.63 MPa and 61.73 MPa after 7 and 28 days, respectively (for C50M50 1.0). The M100 1.0 sample is dominated by aluminosilicate gel, with even the round pores filled with gel, which is a precursor of zeolitic phases. Higher aluminosilicate gel content is closely linked to the compact microstructure and results in high mechanical properties (Figure 3a). As indicated in the study by Karatas et al. [45], the primary reaction product resulting from the activation of metakaolin is a Na_2_O Al_2_O_3_ SiO_2_ H_2_O (N-A-S-H) geopolymer gel, which can be regarded as a zeolite precursor. As illustrated in Figure 5, the incorporation of 100% metakaolin waste precursors results in the formation of N-A-S-H gel.

## 4. Conclusions

The study aimed to develop geopolymer binders using exclusively ceramic brick and metakaolin waste precursor materials, as well as a blend of these two waste types along with different Na_2_SiO_3_/NaOH ratios. The following conclusions can be drawn:

- The utilization of CBW and MKW precursor materials yielded disparate oxide and compound compositions. Therefore, in order to preserve an identical mixture consistency and mixing time, the compositions that were designed used different alkaline solutions to precursor (AS/P) ratios. The utilization of disparate AS/P ratios led to the conclusion that the evaluation of the Na_2_SiO_3_/NaOH ratio’s influence on geopolymer mechanical properties is challenging, as other essential ratios, such as AS/P, W/S, and the total percentage of Na_2_O content, exert a substantial influence on the mechanical properties of hardened geopolymer.
- The utilization of CBW precursor alone within the geopolymer binder system (compositions: C100 0.5; C100 1.0; C100 2.0) has resulted in mediocre compressive strengths (up to 30MPa) when varying Na_2_SiO_3_/NaOH ratios are employed. The compositions in question exhibited the lowest AS/P and W/S ratios and total Na_2_O content, while concurrently demonstrating the highest SiO_2_/Al_2_O_3_ content. This phenomenon is attributed to the fact that CBW particles exhibit a low content of Al_2_O_3_ and a low amorphous phase, both of which are prerequisites for stimulating the geopolymerization reaction at an early age. This results in a significant presence of unreacted particles, as evidenced by XRD and SEM analyses, while an excessive amount of Na_2_O prompts the efflorescence effect. The utilization of a higher intensity curing regime is more probable in inducing secondary geopolymerization and enhancing mechanical properties of CBW-based geopolymer binder.
- The incorporation of blend CBW and MKW precursors in geopolymer binder systems (compositions C50M50 0.5, C50M50 1.0, and C50M50 2.0) has resulted in the enhancement of compressive strength, with values reaching up to 62 MPa at a Na_2_SiO_3_/NaOH ratio of 1.0. This composition exhibited mediocre AS/P and W/S ratios, total Na_2_O content, and SiO_2_/Al_2_O_3_ content. The observed compressive strength is primarily attributed to a relatively optimal CBW and MKW particle distribution, which contains a sufficient number of reactive particles to facilitate adequate geopolymerization reactions. The XRD and FT-IR analyses demonstrated the formation of new phases, namely sodium aluminum silicate hydrate gel, which strengthens the geopolymer matrix and results in favorable mechanical properties.
- The use of MKW precursor in isolation within the geopolymer binder system (compositions M100 0.5, M100 1.0, and M100 2.0) has yielded the maximum compressive strengths. The highest recorded compressive strength of 74.91 MPa after 28 days was attained with a Na_2_SiO_3_/NaOH ratio of 1.0. This composition exhibited the highest AS/P ratio, W/S ratio, and total Na_2_O content, but concurrently demonstrated the lowest SiO_2_/Al_2_O_3_ content. The observed strength growth is attributable to the high reactivity of the MKW particles, which are characterized by substantial Al_2_O_3_ content and a high degree of amorphousness. This inherent reactivity leads to a robust geopolymerization process. The XRD and FT-IR analyses demonstrated the formation of new phases, namely calcium aluminum silicate hydrate (goosecreekite) and N-A-S-H gels. These phases contribute to the formation of a compact matrix, thereby enhancing the material’s compressive strength. Thus, these compositions are strong, but the high AS/P ratio indicates excessive free water in the matrix, leading to high open porosity and brittleness.

The long-term durability of the ceramic brick and metakaolin waste-based geopolymer system remains insufficiently resolved. Sodium silicate and hydroxide are common, but alternatives like sodium carbonate can reduce environmental impact and improve long-term performance when properly balanced. The formation of stable phases through promoting C-(N)-A-S-H gels and zeolite-like phases improves chemical resistance and long-term durability. This change would reduce the open porosity of metakaolin-based geopolymer binder, improving strength and durability.

## Figures and Tables

**Table 1:** Geopolymer binder compositions, %wt.

Notation	Precursor			Alkaline Activator				Essential Ratios					Depth of Plunger Penetration, (mm)
	CBW	MKW	PG	Sodium Silicate (SS)	Sodium Hydroxide (SH)	SS/SH ratio	Na_2_O Content (%)	SiO_2_/Al_2_O_3_	Na_2_O/Al_2_O_3_	H_2_O/Na_2_O	AS/P^1^	W/S^2^	
C100 0.5	100	0	5	11.9	23.9	0.5	5.36	9.46	0.90	12.33	0.36	0.22	19
C100 1.0	100	0	5	17.9	17.9	1.0	4.79	9.68	0.81	13.30	0.36	0.21	22
C100 2.0	100	0	5	23.9	11.9	2.0	4.23	9.89	0.71	14.54	0.36	0.21	24
C50M50 0.5	50	50	5	18.1	36.1	0.5	8.98	5.22	1.03	9.80	0.54	0.32	18
C50M50 1.0	50	50	5	27.1	27.1	1.0	8.23	5.42	0.95	10.33	0.54	0.31	21
C50M50 2.0	50	50	5	36.1	18.1	2.0	7.48	5.61	0.86	10.96	0.54	0.30	23
M100 0.5	0	100	5	27.8	55.6	0.5	12.19	3.46	1.19	9.34	0.83	0.45	18
M100 1.0	0	100	5	41.7	41.7	1.0	11.21	3.68	1.09	9.80	0.83	0.44	22
M100 2.0	0	100	5	55.6	27.8	2.0	10.24	3.89	1.00	10.35	0.83	0.42	25

Here, AS/P^1^—alkali-solution-to-precursor (powder) ratio; W/S^2^—water-to-solids ratio.

**Table 2:** The influence of the precursor type and alkaline activator parameters on the compressive strength of the hardened geopolymers.

Precursor	Mixture Type	Na_2_SiO_3_/NaOH Ratio	NaOH Concentration	Curing Conditions	Highest Comp. Strength, MPa	Reference
metakaolin & fly ash	binder	2.5	8 M	100 °C for 12 h	29.9	[26]
metakaolin & fly ash	binder	2.0	9 M	60 °C for 2 h	25.1	[27]
fly ash & ceramic waste powder	mortar	3.0	14 M	ambient temp.	40.2	[28]
fly ash & ceramic brick waste	mortar	2.5	14 M	ambient temp.	50.0	[29]
metakaolin	binder	1.0	10 M	60 °C for 24 h	16.4	[22]
metakaolin	binder	2.5	14 M	70 °C for 24 h	34.8	[30]
Ceramic clay waste	binder	1.0	10 M	45 °C for 72 h	28.9	[31]
Ceramic waste powder	mortar	2.5	10 M	90 °C for 24 h	27.9	[32]
ceramic brick waste & metakaolin	binder	2.0	13 M	60 °C for 24 h	>60.0	[33]
ceramic brick waste	binder	1.0	10 M	60 °C for 24 h	29.3	current study
metakaolin	binder				74.9	
ceramic brick waste & metakaolin	binder				61.7	

## Data Availability

The original contributions presented in this study are included in the article. Further inquiries can be directed to the corresponding author.
